# A neonatal case of HDR syndrome and a vascular ring with a novel *GATA3* mutation

**DOI:** 10.1038/s41439-019-0087-1

**Published:** 2019-12-23

**Authors:** Moe Kusakawa, Takeshi Sato, Ai Hosoda, Eriko Araki, Yohei Matsuzaki, Yukio Yamashita, Jun Ishihara, Yoshinori Inagaki, Noboru Uchida, Tomohiro Ishii, Tomonobu Hasegawa

**Affiliations:** 1 Department of Pediatrics, Yokohama Municipal Citizen’s Hospital, 56 Okazawa-cho, Hodogaya-ku, Yokohama, Kanagawa Japan; 2 Department of Pediatrics, Keio University School of Medicine, 35 Shinanomachi, Shinjuku-ku, Tokyo Japan; 3 Department of Neonatology, Kanagawa Children’s Medical Center, 2-138-4 Mutsukawa, Minami-ku, Yokohama, Kanagawa Japan

**Keywords:** Mutation, Congenital heart defects, Parathyroid diseases

## Abstract

HDR syndrome (OMIM #146255) is caused by haploinsufficiency of the *GATA3* gene. A vascular ring has not been reported in patients with *GATA3*-associated HDR syndrome. We report a neonatal case of HDR syndrome and a vascular ring that were possibly due to a novel frameshift mutation in the *GATA3* gene.

HDR syndrome is characterized by the triad of hypoparathyroidism, sensorineural deafness, and renal disease [1]. This disorder is caused by haploinsufficiency of the *GATA3* gene related to disruption of the zinc-finger domain of GATA3. A vascular ring, which consists of abnormal blood vessels, is a congenital malformation of the aortic arch and its branches surrounding the trachea and esophagus. To date, a vascular ring has not been reported in patients with *GATA3*-associated HDR syndrome. We report a case of HDR syndrome with a vascular ring in a patient who had a novel *GATA3* mutation.

The proband was the first child of healthy, nonconsanguineous Japanese parents. He was suspected to have a vascular ring by fetal ultrasound evaluation. He was born at full-term, with a birth weight of 2610 g (−0.1 SD) and length of 50.4 cm (+1.6 SD). A contrast computed tomography scan and echocardiography showed that the right aortic arch, aberrant origin of the left subclavian artery, and the left arterial duct formed a vascular ring (Fig. 1). On the 24th day of life, he exhibited generalized convulsions due to hypocalcemia (6.0 mg/dL, reference 9.0–11.0). He also had hyperphosphatemia (10.8 mg/dL, reference 5.0–7.7) and relatively low levels of intact parathyroid hormone (38 pg/mL, reference 10–65), indicating primary hypoparathyroidism. He had a horseshoe kidney and moderate bilateral sensorineural hearing loss. Thus, he was clinically diagnosed with HDR syndrome.

**Fig. 1:**
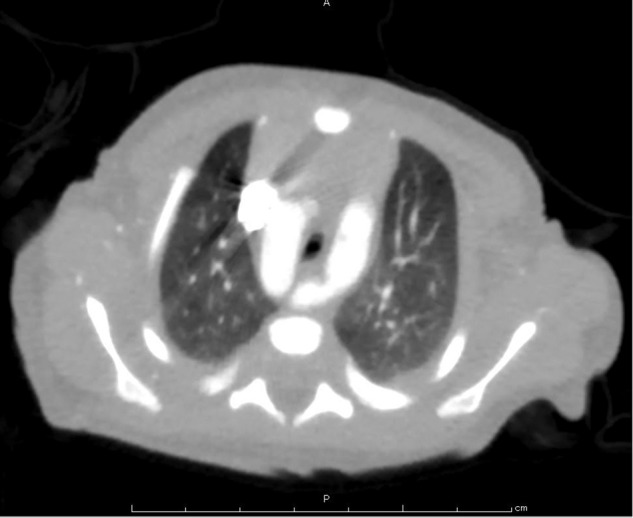
A contrast computed tomography scan on the 3rd day of life. The right aortic arch, aberrant origin of the left subclavian artery, and left arterial duct formed a vascular ring.

We received approval for the genetic test from the institutional review board. After obtaining informed consent from his parents, we extracted genomic DNA from peripheral blood samples from the patient. We amplified all the coding exons and flanking introns of the exons in the *GATA3* gene and performed direct sequencing in both directions on an autosequencer. The sequencing identified a novel heterozygous variant, c.649_653delinsAAA, p.His217Lysfs*86 in the *GATA3* (NM_001002295) gene (Fig. 2). This variant was not found in either the Human Genetic Variation Database or the Exome Aggregation Consortium database.

**Fig. 2:**
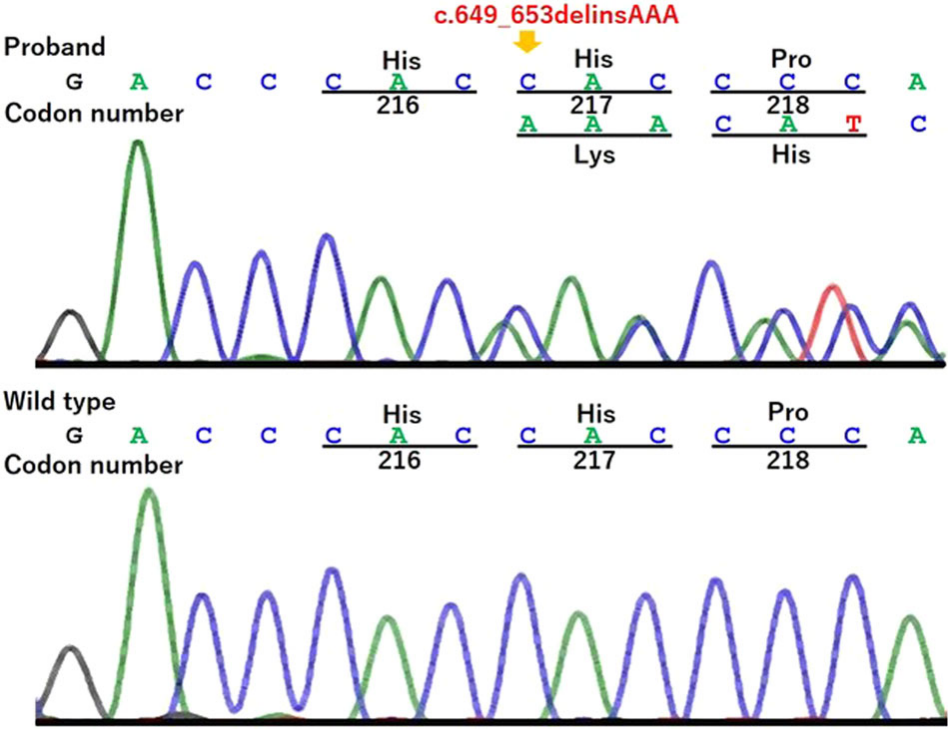
Partial sequence of exon 3 of the *GATA3* gene. The upper panel shows a chromatogram of the proband, who had a heterozygous mutation, c.649_653delinsAAA, p.His217Lysfs*86, which is denoted by an arrow. The lower panel shows a chromatogram of the wild-type sequence.

This is the first case report of HDR syndrome with a concomitant vascular ring. The proband had a novel frameshift mutation in the *GATA3* gene. This frameshift mutation leads to disruption of the zinc-finger domain and probably causes haploinsufficiency of *GATA3*.

The relation between *GATA3* mutations and a vascular ring remains unknown. A previous study showed that, in several dog breeds, single nucleotide polymorphisms in the *TBX1* gene were associated with a persistent right aortic arch, a possible component of a vascular ring [2, 3]. The *Tbx1* gene was shown to be cooperatively regulated by GATA3 and Foxa2 proteins [4]. Another previous study using Gata3 null mice revealed that Gata3 plays an important role in cardiac outflow tract formation [5]. Therefore, Gata3 mutations may contribute to the structural anomalies of the aortic arch. We speculate that a vascular ring, which is not suspected on routine echocardiography and chest radiography, remains undetected in most patients. This is a possible explanation for the lack of reports describing a vascular ring in *GATA3*-associated HDR syndrome. Thus, although the possibility of coincidence cannot be denied, we speculate that haploinsufficiency of *GATA3* contributes to the development of a vascular ring as well as symptoms of HDR syndrome.

## Data Availability

The relevant data from this Data Report are hosted at the Human Genome Variation Database at 10.6084/m9.figshare.hgv.2798
